# Yeast-Based Genetic Interaction Analysis of Human Kinome

**DOI:** 10.3390/cells9051156

**Published:** 2020-05-07

**Authors:** Jae-Hong Kim, Yeojin Seo, Myungjin Jo, Hyejin Jeon, Won-Ha Lee, Nozomu Yachie, Quan Zhong, Marc Vidal, Frederick P. Roth, Kyoungho Suk

**Affiliations:** 1 Department of Pharmacology, Brain Science and Engineering Institute, and Department of Biomedical Sciences, BK21 Plus KNU Biomedical Convergence Program, School of Medicine, Kyungpook National University, Daegu 41944, Korea; kim86217@nate.com (J.-H.K.); seoyodini@naver.com (Y.S.); jinyhoi11@naver.com (M.J.); hyejin7432@hanmail.net (H.J.); 2 School of Life Sciences, Brain Korea 21 Plus KNU Creative BioResearch Group, Kyungpook National University, Daegu 41566, Korea; whl@knu.ac.kr; 3 Donnelly Centre and Departments of Molecular Genetics and Computer Science, University of Toronto and Lunenfeld-Tanenbaum Research Institute, Sinai Health System, Toronto, ON M5G 1X5, Canada; nzmyachie@gmail.com (N.Y.); fritz.roth@gmail.com (F.P.R.); 4 Department of Biological Sciences, Wright State University, Dayton, OH 45435, USA; zhongquan99@gmail.com; 5 Center for Cancer Systems Biology (CCSB) and Department of Cancer Biology, Dana-Farber Cancer Institute, Boston, MA 02215, USA; Marc_Vidal@dfci.harvard.edu

**Keywords:** yeast, kinase, genetic interaction, network, bar-seq

## Abstract

Kinases are critical intracellular signaling proteins. To better understand kinase-mediated signal transduction, a large-scale human–yeast genetic interaction screen was performed. Among 597 human kinase genes tested, 28 displayed strong toxicity in yeast when overexpressed. *En masse* transformation of these toxic kinase genes into 4653 homozygous diploid yeast deletion mutants followed by barcode sequencing identified yeast toxicity modifiers and thus their human orthologs. Subsequent network analyses and functional grouping revealed that the 28 kinases and their 676 interaction partners (corresponding to a total of 969 genetic interactions) are enriched in cell death and survival (34%), small-molecule biochemistry (18%) and molecular transport (11%), among others. In the subnetwork analyses, a few kinases were commonly associated with glioma, cell migration and cell death/survival. Our analysis enabled the creation of a first draft of the kinase genetic interactome network and identified multiple drug targets for inflammatory diseases and cancer, in which deregulated kinase signaling plays a pathogenic role.

## 1. Introduction

Kinases catalyze the transfer of phosphate groups from high-energy phosphate-donating molecules to specific substrates via a process known as phosphorylation. Proteins, lipids and carbohydrates can be substrates of the phosphorylation process. The phosphorylation states of these molecules affect their activity and ability to bind other molecules, thereby regulating cell signaling, metabolism and other important cellular pathways. As such, kinases constitute a major component of intracellular signaling pathways. As the single most important protein family in signal transduction, they were extensively exploited as a drug target for a variety of cancers and immune/inflammatory disorders [1,2]. Nevertheless, the elucidation of numerous kinase-signaling pathways and the crosstalk between them remains a formidable challenge, necessitating intense investigations currently in progress worldwide. As one-way to address kinase signaling pathways, protein-protein interaction and network-based systems approaches have been previously used. Several recent studies addressed this issue by building and exploring an integrated human kinase network at the genome level [3]; for example, large-scale discovery of kinase substrates [4], kinase inhibitors [5] and functional analysis of kinase and phosphatase overexpression [6]. Yeast, as a genetically tractable eukaryotic organism [7], is also used for systems biology studies. For example, the *Saccharomyces cerevisiae* genome has been sequenced in its entirety [8] and genome-scale genetic interactions have been profiled [9,10]. Genetic interactions between yeast and human disease genes have been previously used to predict the pathological functions of disease genes and to understand disease mechanisms [11,12,13,14].

In this study, to better understand kinase signaling pathways and to uncover new drug targets related to these pathways, we performed a genome-wide human–yeast genetic interaction screen for yeast deletions that can relieve the effects of toxic human kinase expression. Screening was carried out in multiplexed pool format, which is more efficient than an array format for the large-scale analysis of genetic interactions. Networks and subnetworks of genetic interactions constructed from select human kinases provide a better understanding of the molecular basis of intracellular kinase signaling pathways and a platform for further investigation of the entire kinase interactome.

## 2. Materials and Methods

### 2.1. Yeast Strains, Media, Plasmids and Virus

BY4742 (Mat α; *his3Δ1*; *leu2Δ0*, *lys2Δ0*; *ura3Δ0*) was used as a wild-type yeast strain in this study. The Homozygous Diploid Complete Set of Yeast Deletion Clones and Homozygous Diploid Yeast Deletion Pools was purchased from Invitrogen (Carlsbad, CA, USA). Yeast cells were grown in rich medium (YPD) or synthetic medium lacking leucine and containing 2% glucose (SD-Leu), raffinose (SRaf-Leu) or galactose (SGal-Leu). Gateway entry clones of full-length human kinase cDNAs were derived from the hORFeome V8.1 entry clone collection (http://horfdb.dfci.harvard.edu). The Gateway LR reaction was used to shuttle kinase cDNAs into pAG425GAL-ccdB (Addgene, Cambridge, MA, USA) [15] for yeast expression. All plasmids were 2-μm-based and under the control of the *GAL1* promoter. All constructs were verified by Sanger sequencing. For functional studies in mammalian cells or mice, the Gateway LR reaction was used to shuttle kinase open reading frames (ORFs) into pDS-GFP-XB (Invitrogen) destination vectors.

### 2.2. Yeast Transformation and Spotting Assays

Kinase cDNAs in pAG425GAL (yeast destination vector) were transformed into BY4742 or homozygous diploid deletion strains. All yeast strains were grown at 30 °C according to the standard protocol. We used the LiAc/SS carrier DNA/PEG method to transform yeast with plasmid DNA as previously described [16]. For spotting assays, yeast cells were grown overnight at 30 °C in SRaf-Leu media. Cultures were serially diluted and spotted onto SD-Leu or SGal-Leu medium and grown at 30 °C for 3–5 days. Two independent transformants were tested in the spotting assays, which gave similar results.

### 2.3. Human–Yeast Genetic Interaction Screen

Kinase cDNAs were transformed into homozygous diploid yeast deletion pools containing 4653 individual deletion clones. Transformants were selected by incubating cells in 5 mL SD-Leu medium. To determine the transformation efficiency, 0.1% of the cells (5 μL) were plated onto SD-Leu agar plates. Approximately 50–100 individual transformants were obtained, indicating 10- to 20-fold coverage of the deletion library. Transformants were incubated in SD-Leu medium for 16 hr. The cells were washed twice with PBS and then incubated in SGal-Leu medium for 2 days. Cells remaining in glucose-containing SD-Leu medium were used as a control. Genomic DNA was isolated from cells harvested after pooled growth. Each 20-mer UPTAG barcode was amplified using composite primers comprised of the sequence of the indexing tag and the sequence of the common barcode primers: 5′-G*NNNNNN***GATGTCCACGAGGTCTCT**-3′ (forward) and 5′-C*NNNNNN***GTCGACCTGCAGCGTACG**-3′ (reverse). The 5′ portion (italics) indicates the variable sequence, which represents the 6-mer indexing tag used for multiplexing. The 3′ portion (bold) represents the common primer flanking the UPTAG barcode; it is required to amplify the yeast barcodes. PCR amplification was carried out at an annealing temperature of 55 °C for 30 cycles using a DNA Engine Tetrad Peltier Thermal Cycler (MJ Research, Waltham, MA, USA). The PCR products were gel-purified from 4% agarose gels. Equal volumes of normalized DNA were then pooled in one tube and sequenced using a Genome Analyzer (Illumina, San Diego, CA, USA) according to the manufacturer’s protocols.

### 2.4. Analysis of Illumina Sequencing

To analyze the barcode sequencing (Bar-seq) data, all counts were rescaled, dividing each count by the average of barcode counts across the pooled experiment for each given kinase. Rescaled barcode counts were converted into Z-scores for each yeast gene by subtracting the mean for that gene across all kinase experiments and dividing by the standard deviation for that gene across all kinase experiments.

### 2.5. Bioinformatics Analysis and Network Construction

Ingenuity Pathway Analysis (IPA), Database for Annotation, Visualization and Integrated Discovery (DAVID; http://david.abcc.ncifcrf.gov/) [17] and STRING: functional protein association networks (http://string-db.org) [18,19] were used to determine the biologic function categories of kinases and their interaction partners and to derive networks of genetic interactions and protein–protein interactions. Cytoscape was used to draw network graphs.

### 2.6. Availability of Data and Materials

The Bar-seq data in this study were submitted to the Sequence Read Archive (SRA; https://www.ncbi.nlm.nih.gov/sra/) under accession number SRP113343.

## 3. Results

### 3.1. Selection of 28 Kinase Genes that are Toxic when Overexpressed in Yeast

**Figure 1:**
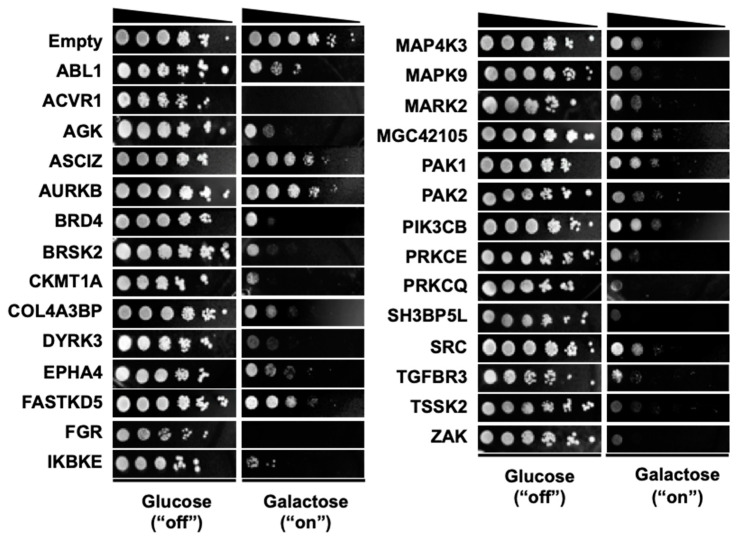
Overexpression of selected kinase genes induces toxicity in yeast. In total, 597 kinase open reading frames (ORFs) were cloned under the control of a galactose-inducible promoter in pAG425 vectors. Empty vector or pAG425GAL-kinases were individually transformed into yeast (BY4742 wild-type strain) and transformants were spotted onto SD-Leu agar plates (kinase expression “off”) or SGal-Leu agar plates (kinase expression “on”). Shown are tenfold serial dilutions starting with an equal number of cells expressing the 28 toxic kinase genes. Non-toxic kinase genes are not shown.

The mapping of gene or protein interaction networks for disease genes improves the understanding of disease mechanisms [20,21]. Functional analysis of kinase signaling pathways can be achieved by mapping genetic interaction networks for kinase genes. Here we sought genes for which perturbation modifies the activity of human kinases. Because genetic interaction screens can be efficiently carried out in yeast, we first sought human kinases that, when expressed in yeast, produced a toxicity phenotype for which modifier genes may be subsequently identified. A total of 597 human kinase cDNAs were cloned into yeast expression vector pAG425Gal-ccdB, enabling their inducible overexpression in yeast. Overexpression of kinases is often associated with an increase in the kinase activity in vitro and in vivo and routinely used to enhance the kinase activity in the experimental settings [22,23,24]. Spotting assays were performed to determine the toxicity of these human kinases in yeast (BY4742 wild-type strain). Of the 597 human kinase genes tested (Supplementary Table S1), 28 kinase cDNAs exhibited strong toxicity when expressed in the BY4742 wild-type strain (Figure 1 and Table 1).

The DNA sequences of the 28 yeast expression clones were verified by Sanger sequencing. The relevance of these kinases in human diseases was next analyzed. Among the 28 kinases, most were associated with cancer and 11 kinases were associated with glioma, based on information from DisGeNET (http://www.disgenet.org).

### 3.2. High-Throughput Identification of Human Kinase–Yeast Genetic Interactions

**Figure 2:**
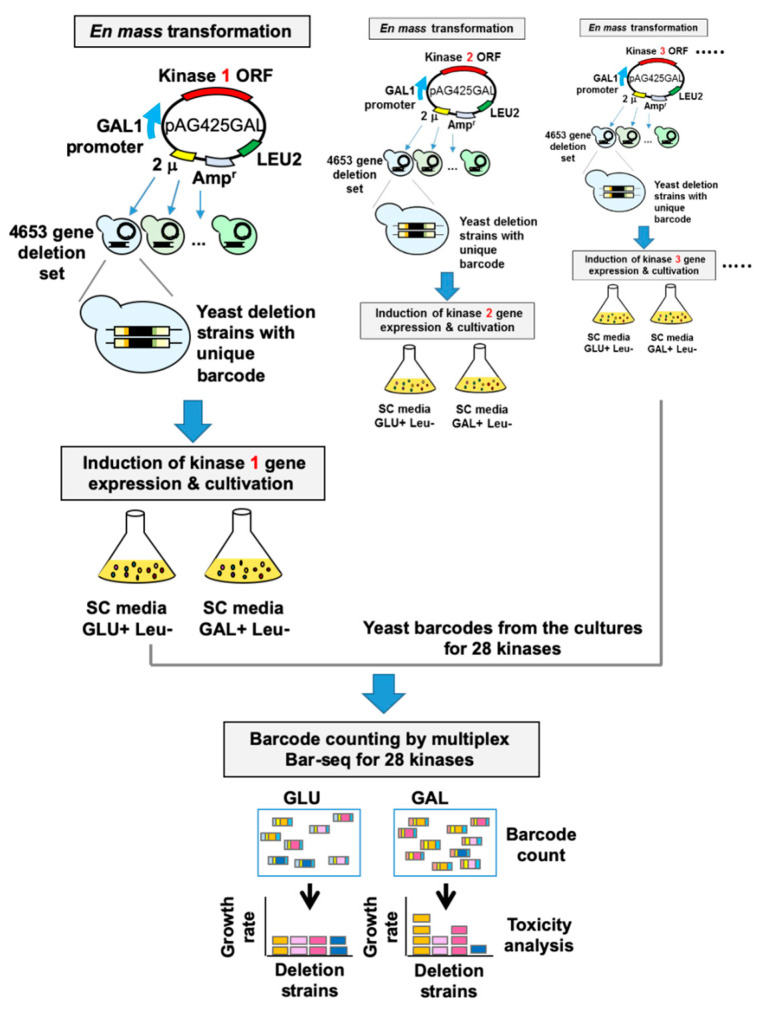
Flowchart of the yeast genetic interaction screen. The 28 toxic kinase genes were transformed individually into a pool of 4653 yeast homozygous deletion strains containing a 20-bp DNA barcode sequence. Transformants were selected in SD-Leu medium and then were resuspended in SGal-Leu medium and incubated for 2 days to induce the expression of kinase genes under the control of the *GAL1* promoter. Genomic DNA was separately isolated from cells harvested after pooled culture in the presence of GLU or GAL. Barcodes were amplified from genomic DNA with multiplexed primers containing distinct combinations of two different tags for each kinase gene. Equal amounts of DNA amplified for each kinase gene were pooled and subjected to multiplex barcode sequencing using an Illumina Genome Analyzer. Next-generation sequencing data were then analyzed for barcode counting, which was used to screen kinase–yeast genetic interactions.

The 28 kinases that were highly toxic in yeast were subjected to further study. To identify human–yeast genetic interactions for these genes, we performed a genome-wide multiplexed pooled screen to identify genetic interactions on the basis of toxicity modification (Figure 2). In this screen, individual kinase genes were first introduced into a pool of 4653 homozygous diploid yeast deletion mutants such that each mutant harbors unique barcode sequences flanking the deletion locus [25]. Second, kinase gene expression was induced by growth of yeast on galactose media. Each yeast deletion pool, expressing a single human kinase gene, was cultured individually. Third, yeast barcodes were amplified en masse from deletion pool cultures for each individual kinase gene. Finally, yeast barcode abundances were quantified using multiplexed next-generation sequencing (Bar-seq) to identify fitness values of human kinase–yeast genetic interactions [26]. The relative abundance of each yeast barcode is a proxy for differential growth of the corresponding deletion strain, which, when compared between different kinase overexpression experiments, allowed us to detect modulation of kinase gene toxicity in the absence of a specific yeast gene [27,28].

For each of 4,653 yeast deletions, we identified the relative abundance of each deletion strain after selection in the presence of each of 28 kinase genes. We obtained Z-scores for each pairing of a yeast deletion and human kinase, standardizing relative abundance across the kinase experiments (Supplementary Table S2). Pairs with Z-score > 1.96 were identified as toxicity suppressors and those with Z-score < −1.96 as enhancers (Supplementary Table S3). Suppression or enhancement of kinase toxicity by deletion of a specific yeast gene in the large-scale screen was validated by yeast spotting assay using individual yeast deletion strains. For this, several yeast/human gene pairs were selected from among suppressors, enhancers or non-modifiers for five kinases, and modification of individual kinase toxicity was tested in the selected yeast deletion strains. The results of the individual spotting assay and the genome-wide screen showed an average agreement of 82.9% in the suppressor group, 33.3% in the non-modifier group, and 43.3% in the enhancer group (Table 2).

**Figure 3:**
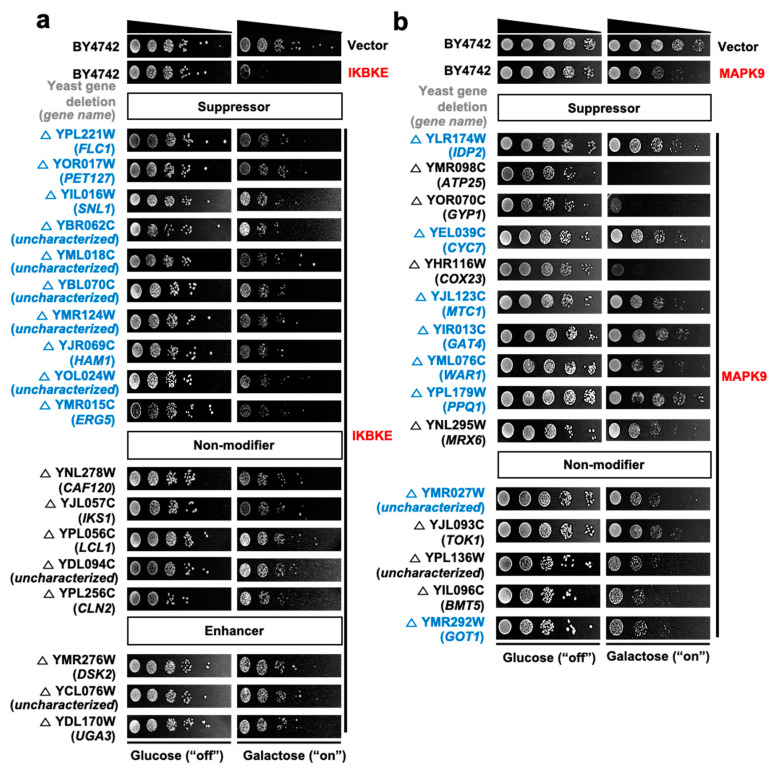

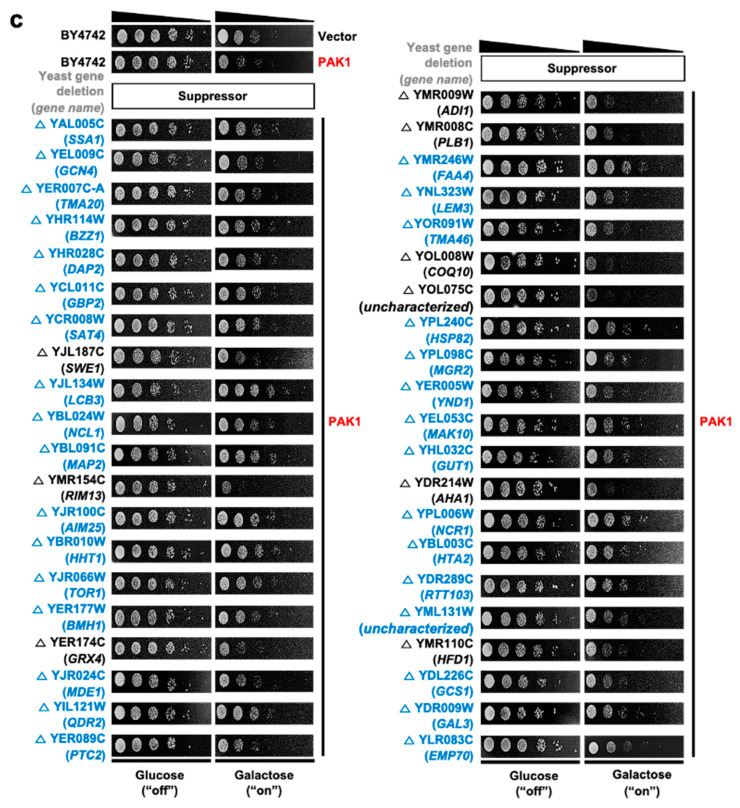

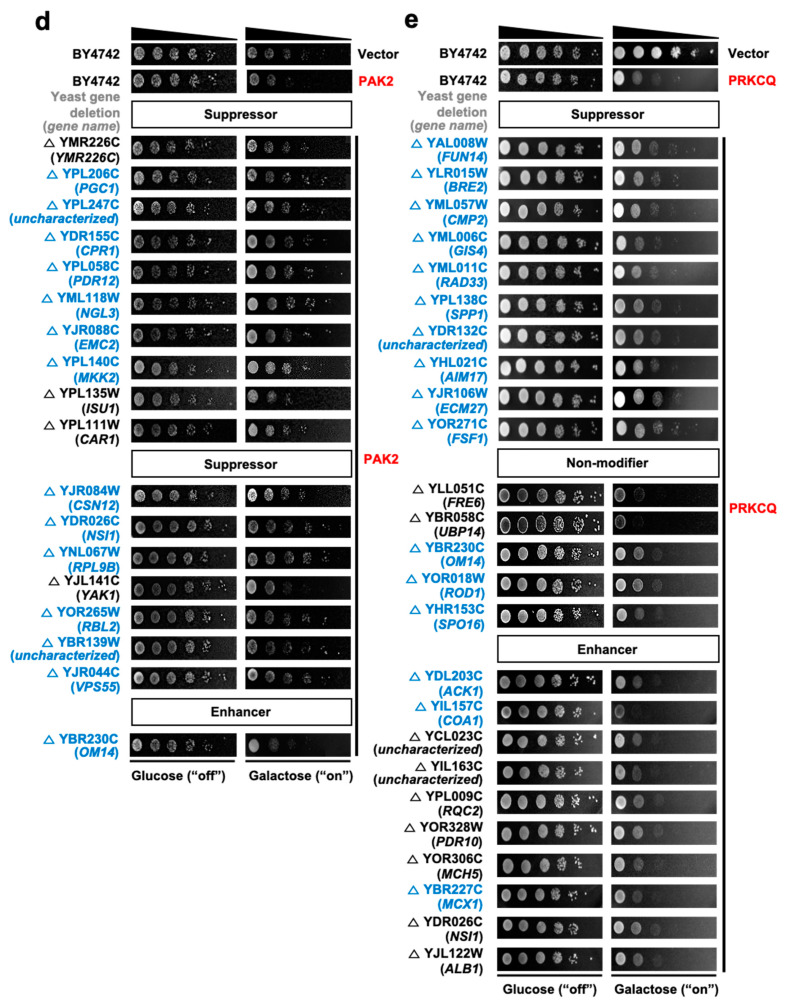
Validation of toxicity modifiers by yeast spotting assays. From the Bar-seq data analysis and Z-score distributions, three groups of yeast genes were chosen for spotting assays: group 1, toxicity suppressors; group 2, non-modifiers; and group 3, toxicity enhancers. Toxicity modifiers for five kinases (IKBKE, MAPK9, PAK1, PAK2 and PRKCQ) were tested by yeast spotting assays. (**a**) Suppressors, non-modifiers and enhancers for IKBKE; (**b**) suppressors and non-modifiers for MAPK9; (**c**) suppressors for PAK1; (**d**) suppressors and enhancers for PAK2; (**e**) suppressors, non-modifiers and enhancers for PRKCQ. For each kinase, 10 suppressors, 5 non-modifiers and 10 enhancers were tested. If the total number of toxicity modifiers was less than 5 for non-modifiers or 10 for enhancers, all modifiers were tested. For PAK1 and PAK2, additional suppressors were tested. Deletion strains in blue indicate consistent results between Bar-seq-based genetic interactions and individual spotting assays. pAG425GAL-ccdB was used as the empty vector control. BY4742 is the wild-type yeast strain.

Representative images of the kinase spotting assay for validation are shown in Figure 3. The average agreement was relatively low for the non-modifier and enhancer groups, possibly because query gene-induced toxicity was too strong to reliably distinguish toxicity enhancers from non-modifiers. Therefore, we restricted subsequent attention to genetic interactions between human kinases and yeast genes such that a given yeast gene deletion suppresses the yeast toxicity of a given human kinase gene.

### 3.3. Construction of Human Kinase Genetic Interaction

**Figure 4:**
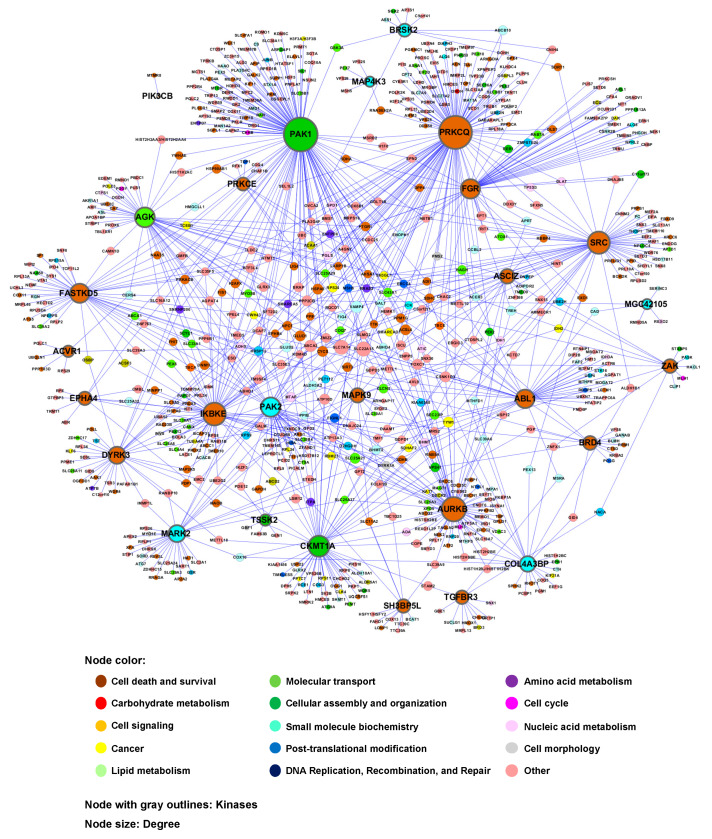
Human–yeast genetic interaction network: kinase interactome network. Human orthologs were identified for yeast genes, the deletion of which suppressed the toxicity of the 28 kinases. A network view of the genetic interactions between these human orthologs and kinases was generated using Cytoscape. The kinase interactome network encompasses the 28 kinases and their 969 genetic interactions. The node color corresponds to the biologic function category to which each gene belongs. The node size is proportional to the number of genetic interactions. Nodes with gray outlines indicate the 28 kinases.

To identify the genetic interactome of human kinases, human orthologs of the yeast toxicity suppressors were identified using the Karolinska Institute’s InParanoid Database (http://inparanoid.sbc.su.se) [29] (Supplementary Table S4). A genetic interaction network between human kinases and other human genes was constructed using the human orthologs of the yeast toxicity suppressors for the 28 kinase genes (Figure 4). This network also revealed the relationships among the 28 kinase genes. IPA-based protein-protein interactions [30] were also included in the second version of the network (Supplementary Figure S1).

Thus, a genome-wide human–yeast genetic interaction screen followed by a search for the human orthologs of the yeast toxicity suppressors provided a “first draft” of a kinase interactome network for a subset of human kinase genes. The number of genetic interaction partners for each kinase is shown in Table 3.

**Figure 5:**
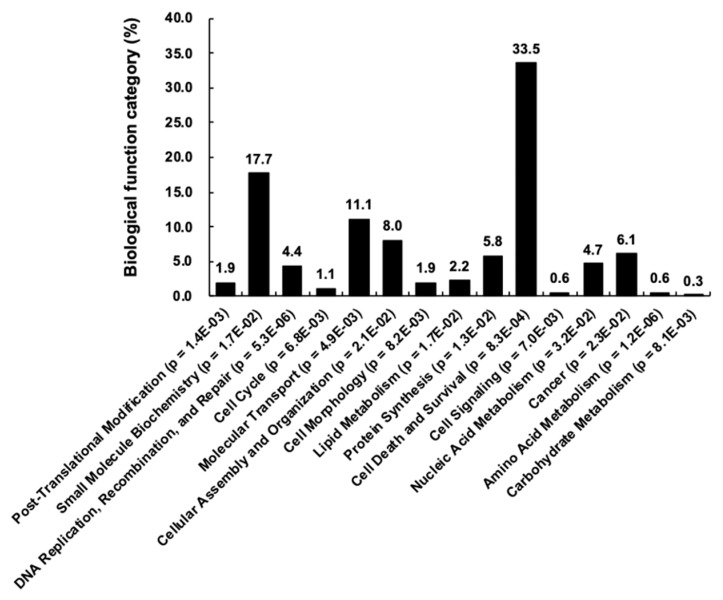
Gene ontology classification. The graph shows biologic function categories of the kinase interactome (28 kinases and 676 genetic interaction partners). The y-axis indicates the percentage in each category.

Analysis of the biologic function category revealed that the 28 kinases and their 676 interaction partners (corresponding to a total of 969 genetic interactions) are enriched in cell death and survival (34%), small-molecule biochemistry (18%) and molecular transport (11%), among others (Figure 5). Because glioma was one of the highly enriched diseases associated with the 28 kinases, cellular behaviors related to glioma, such as cell migration and cell death/survival, were used to derive a subnetwork of relevant genes (Supplementary Figure S2). In the subnetworks, several kinases, such as ABL1, ACVR1, EPHA4, MAPK9, PAK1 and SRC, were commonly associated with glioma, cell migration and cell death/survival. These results were not unexpected, because the genetic interactions were based on the yeast toxicity. Nevertheless, our findings provide insights into complex kinase networks regulating intracellular signaling pathways and suggest kinases and their interaction partners as druggable targets for the therapy of cancer and other diseases, in which kinases play critical roles.

## 4. Discussion

A large-scale human–yeast genetic interaction screen was performed to better understand kinase pathways. Among 597 human kinase genes tested, 28 genes were highly toxic when overexpressed in yeast (Figure 1 and Table 1). A “modifier genetics” approach was applied to these human kinases, in which kinase toxicity was modified by specific deletion of yeast genes. The toxicity modifiers identified in a genome-wide yeast-based phenotypic screen were used to define human–yeast genetic interactions. In our analysis, toxicity suppressors more accurately predicted genetic interactions than toxicity enhancers. Human orthologs of yeast toxicity modifiers were used to construct a kinase interactome network. This approach efficiently identified 969 pairs of genetic interactions for 28 kinases, which were partially validated by individual yeast spotting assays. Although the current kinase interactome network is limited to the 28 kinases that showed strong toxicity in yeast, this approach provides a platform for constructing the entire kinase network in the future. The current kinase network also serves as a basis for a better molecular understanding of kinase signaling pathways relevant to human health and disease.

In this study, kinase gene-induced yeast toxicity formed the phenotypic basis for a genome-wide screen of human–yeast genetic interactions, involving en masse transformation of toxic kinase genes into a barcoded yeast deletion library and subsequent multiplexed barcode sequencing. Although this method was successful in simultaneously identifying genome-wide human–yeast genetic interactions for 28 kinase genes in a pooled and multiplexed format, this screen is not applicable to kinase genes that are not toxic in yeast. This limitation can be overcome by using different growth conditions, higher expression levels of human kinase genes or other yeast phenotypes, such as cell morphology, as a basis for the screen. The yeast toxicity phenotype was used in this study as a starting point to analyze genetic interactions. Moreover, it remains to be determined to what extent genetic interaction discovered in yeast based on the fitness/toxicity of human kinases would be relevant in human. Among the genetic interactions identified, not all leads from the yeast toxicity screen may be relevant in mammalian cells because of the differences in the mechanisms underlying the modification of yeast toxicity and mammalian cell phenotypes. Despite these limitations, current results of yeast toxicity-based screen provide testable hypotheses relating to human kinases and their biologic functions.

The use of galactose to induce the expression of the human kinases in the deletion strains collection may have had confounding effects on the growth of deletion strains. As galactose is first converted to glucose through the Leloir pathway [31] to be metabolized by yeast, different metabolism between glucose and galactose may have influenced the growth of deletion strains, thereby resulting in the differences in the abundance of a given deletion strain in the control (glucose) versus treatment (galactose) conditions. This would affect the Bar-seq data and subsequent network analysis. Thus, our data should be interpreted with caution, in terms of pathways and subnetworks related to metabolism.

The present study was designed to address the entire human kinome and their interactome networks, in contrast to recent studies that focused on specific kinases and their interactome, such as Lemur Tyrosine Kinase 2 (LMTK2) [32], c-Jun N-terminal kinases (JNKs) [33], MET receptor tyrosine kinase [34], LRRK2 [35] and DAP-kinase (DAPK) [36]. To understand and exploit the kinase signaling in broader perspective, a new paradigm in kinome studies is necessary, such as large-scale discovery of substrates of the human kinome [4], quantitative assessment of overexpression effects of human kinome and phosphatome on cell signaling [6] and protein-protein interaction-based drug design of kinase inhibitors [5].

In summary, a genome-wide genetic screen for interactions between human kinase genes and yeast genes provided a first draft of the kinase interactome, serving as the basis for future investigations of kinase pathways. The kinase network identified in this study may enable a better understanding of kinase signaling pathways under diverse physiological conditions and provides potential drug targets for pathological conditions such as inflammatory diseases, cancer and other disorders, in which kinases play an important role.

## Figures and Tables

**Table 1:** List of human kinase genes that induce strong toxicity in yeast.

No.	Gene Symbol	UniProt ID	Common Name	Note ^a^
1	ABL1	P00519	c-Abl oncogene 1, non-receptor tyrosine kinase	Tyr
2	ACVR1	Q04771	Activin receptor type-1	Ser/Thr
3	AGK	Q53H12	Acylglycerol kinase, mitochondrial	Lipid
4	ASCIZ	D3DUL0	ATM/ATR-Substrate Chk2-Interacting Zn2+-finger protein, isoform CRA_b	Putative kinase
5	AURKB	Q96GD4	Aurora kinase B	Ser/Thr
6	BRD4	O60885	Bromodomain-containing protein 4	Putative kinase
7	BRSK2	Q8IWQ3	BR serine/threonine kinase 2	Ser/Thr
8	CKMT1A	P12532	Creatine kinase U-type, mitochondrial	Creatine
9	COL4A3BP	Q9Y5P4	Collagen type IV alpha-3-binding protein	Putative kinase
10	DYRK3	O43781	Dual specificity tyrosine-phosphorylation-regulated kinase 3	Ser/Thr, Tyr
11	EPHA4	P54764	Ephrin type-A receptor 4	Tyr
12	FASTKD5	Q7L8L6	FAST kinase domain-containing protein 5	Putative kinase
13	FGR	P09769	Feline Gardner-Rasheed sarcoma viral oncogene homolog	Tyr
14	IKBKE	Q14164	Inhibitor of nuclear factor kappa-B kinase subunit epsilon	Ser/Thr
15	MAP4K3	Q8IVH8	Mitogen-activated protein kinase kinase kinase kinase 3	Ser/Thr
16	MAPK9	P45984	Mitogen-activated protein kinase 9	Ser/Thr
17	MARK2	Q7KZI7	MAP/microtubule affinity-regulating kinase 2	Ser/Thr
18	MGC42105	A0A024R049	Uncharacterized protein	Putative kinase
19	PAK1	Q13153	p21 protein (Cdc42/Rac)-activated kinase 1	Ser/Thr
20	PAK2	Q13177	p21 protein (Cdc42/Rac)-activated kinase 2	Ser/Thr
21	PIK3CB	P42338	Phosphatidylinositol 4,5-bisphosphate 3-kinase catalytic subunit beta isoform	Lipid
22	PRKCE	Q02156	Protein kinase C epsilon type	Ser/Thr
23	PRKCQ	Q04759	Protein kinase C theta	Ser/Thr
24	SH3BP5L	Q7L8J4	SH3 domain-binding protein 5-like	Putative kinase
25	SRC	P12931	Proto-oncogene tyrosine-protein kinase	Tyr
26	TGFBR3	Q03167	Transforming growth factor beta receptor type 3	Ser/Thr
27	TSSK2	Q96PF2	Testis-specific serine/threonine-protein kinase 2	Ser/Thr
28	ZAK	Q9NYL2	Mitogen-activated protein kinase kinase kinase 20	Ser/Thr

^a^ Characteristics of kinases, such as phosphorylation residues and their substrates, are shown. Kinase activity has not been directly demonstrated for putative kinases.

**Table 2:** Summary of the results of yeast spotting assays.

**Kinase**	**Toxicity Suppressors**		
	**Suppressor/tested**	**Consistency**	**Average**
IKBKE	10/10	100%	82.9%
MAPK9	6/10	60%	
PAK1	32/41	78%	
PAK2	13/17	76.5%	
PRKCQ	10/10	100%	
**Kinase**	**Non-Modifiers**		
	**Non-modifier/tested**	**Consistency**	**Average**
IKBKE	0/5	0%	33.3%
MAPK9	2/5	40%	
PAK1	NT ^a^	NT	
PAK2	NT	NT	
PRKCQ	3/5	60%	
**Kinase**	**Toxicity Enhancers**		
	**Enhancer/tested**	**Consistency**	**Average**
IKBKE	0/3	0%	43.3%
MAPK9	- ^b^	-	
PAK1	-	-	
PAK2	1/1	100%	
PRKCQ	3/10	30%	

^a^ NT, not tested; ^b^ there is no enhancer for this kinase.

**Table 3:** The number of genetic interaction partners for each kinase.

No.	Kinase	Z-Score (>1.96) ^a^	Presence of Human Ortholog ^b^
1	ABL1	124	41
2	ACVR1	23	6
3	AGK	138	49
4	ASCIZ	60	18
5	AURKB	203	75
6	BRD4	40	14
7	BRSK2	27	9
8	CKMT1A	164	56
9	COL4A3BP	54	24
10	DYRK3	120	33
11	EPHA4	35	11
12	FASTKD5	138	45
13	FGR	134	46
14	IKBKE	189	66
15	MAP4K3	22	5
16	MAPK9	45	17
17	MARK2	103	32
18	MGC42105	10	5
19	PAK1	402	131
20	PAK2	135	45
21	PIK3CB	14	1
22	PRKCE	31	9
23	PRKCQ	322	126
24	SH3BP5L	25	12
25	SRC	211	63
26	TGFBR3	33	11
27	TSSK2	23	6
28	ZAK	53	13

^a^ The number of genetic interaction partners with a Z-score higher than 1.96, which corresponds to yeast toxicity suppressors. ^b^ The number of genetic interaction partners with a human ortholog.

## Supplementary Materials

The following are available online at https://www.mdpi.com/2073-4409/9/5/1156/s1. Figure S1. Human kinase–yeast interactome network. Figure S2. Subnetwork of human kinase–yeast genetic interactions. Table S1. List of human kinases. Table S2. Barcode counts of yeast deletion strains obtained from high-throughput sequencing. Table S3. List of toxicity modifiers for the 28 human kinases. Table S4. Human orthologs of the toxicity suppressors.
