# Supplementary material for: Yeast-Based Genetic Interaction Analysis of Human Kinome
Source: Cells. 2020 May 7;9(5):1156. doi: 10.3390/cells9051156 (PMC7291280; doi:10.3390/cells9051156)
Supplement: Supplementary file 1 [file cells-09-01156-s001.zip › Supplementary Figures.pdf]

Figure S1

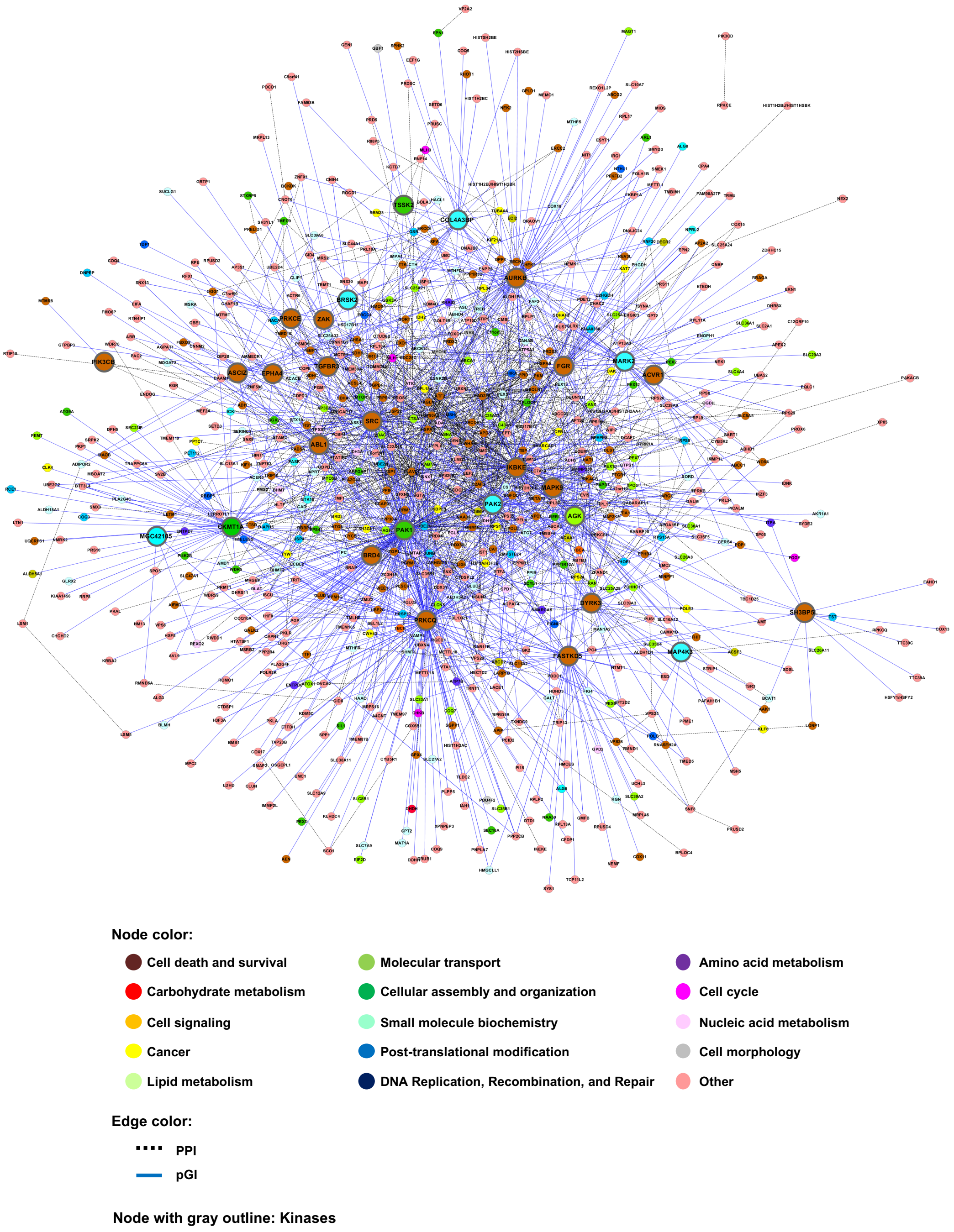

Figure S1. Human kinase-yeast interactome network. Human orthologs of yeast genes, the deletion of which suppressed the toxicity of the 28 kinase ORFs, were identified. A network view of these orthologs was generated using Cytoscape. The color of the node corresponds to the biological function category to which the gene belongs. The color of an edge indicates the type of interaction (PPI, protein-protein interaction, black dotted line; pGI, predicted genetic interaction, blue line).

Figure S2.

a Kinase network

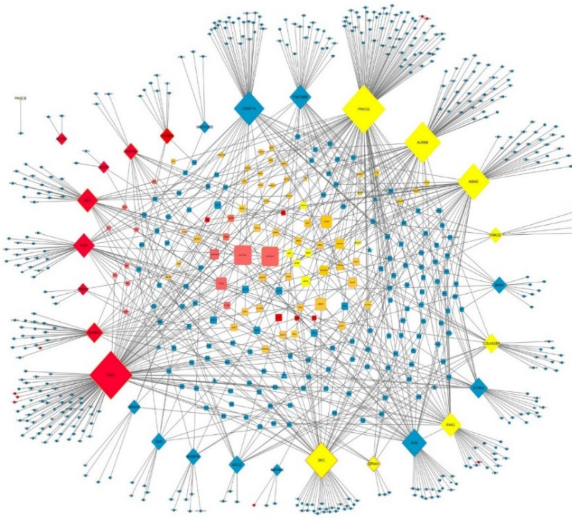

- Cell migration-related kinases
- Glioma-related kinases
- Other kinases
- Interaction partners of cell migration-related kinases
- Interaction partners of glioma-related kinases
- Cell migration-related interaction partners
- Glioma-related interaction partners
- Other interaction partners
- Cell migration and glioma-related kinases

b Subnetwork of cell migration-related kinases

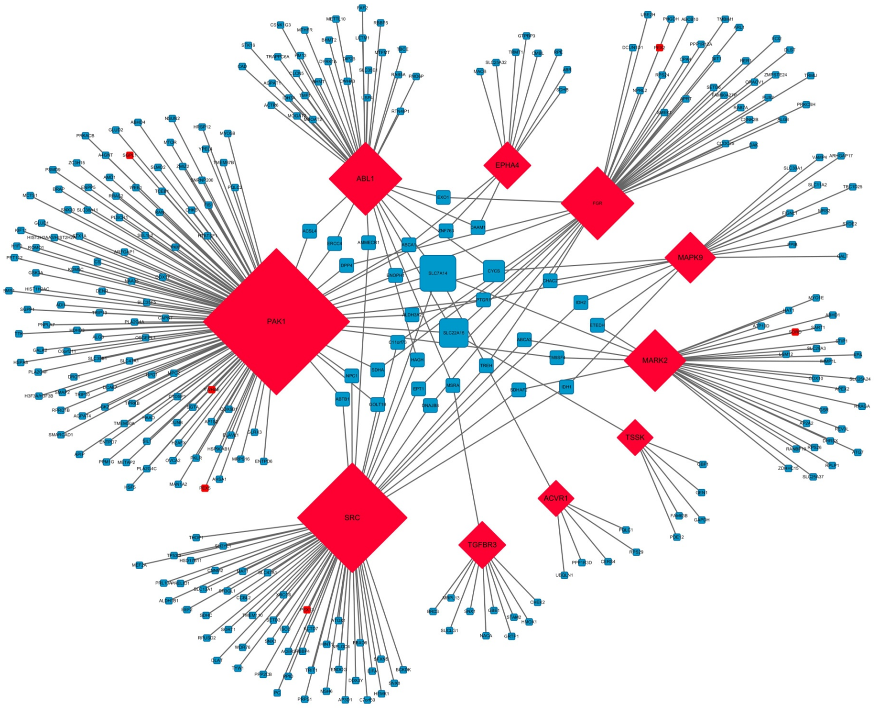

- Cell migration-related kinases
- Cell migration-related interaction partners
- Other interaction partners

c **Subnetwork of glioma and cell migration-related kinases**

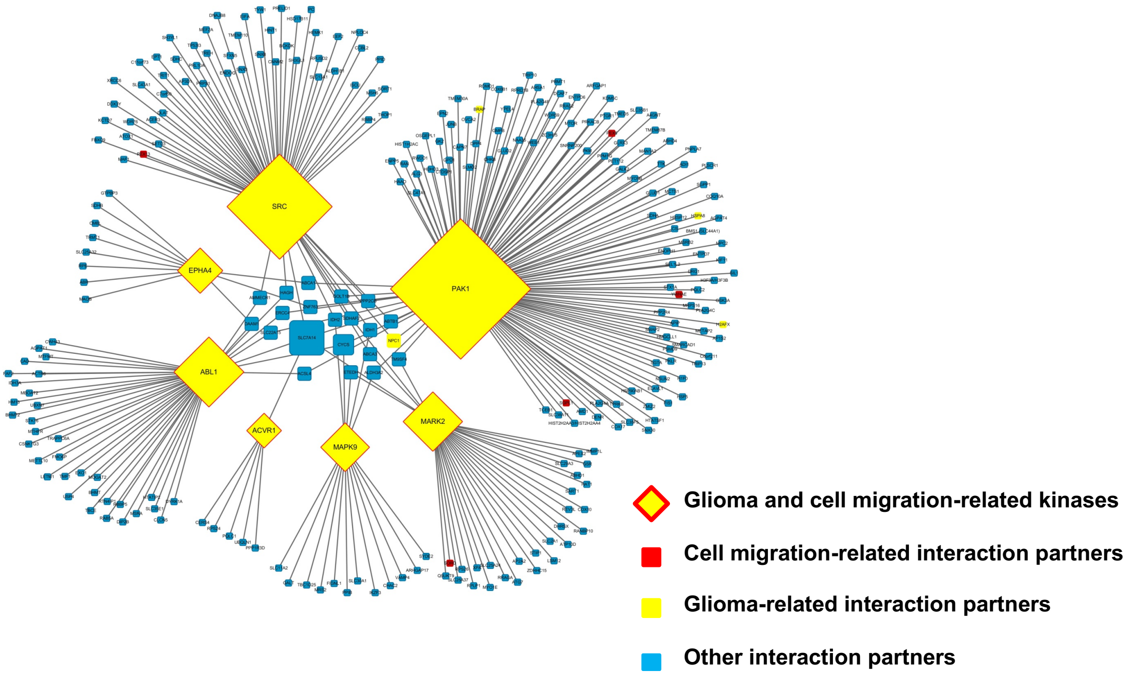

d **Subnetwork of glioma and cell death/survival-related kinases**

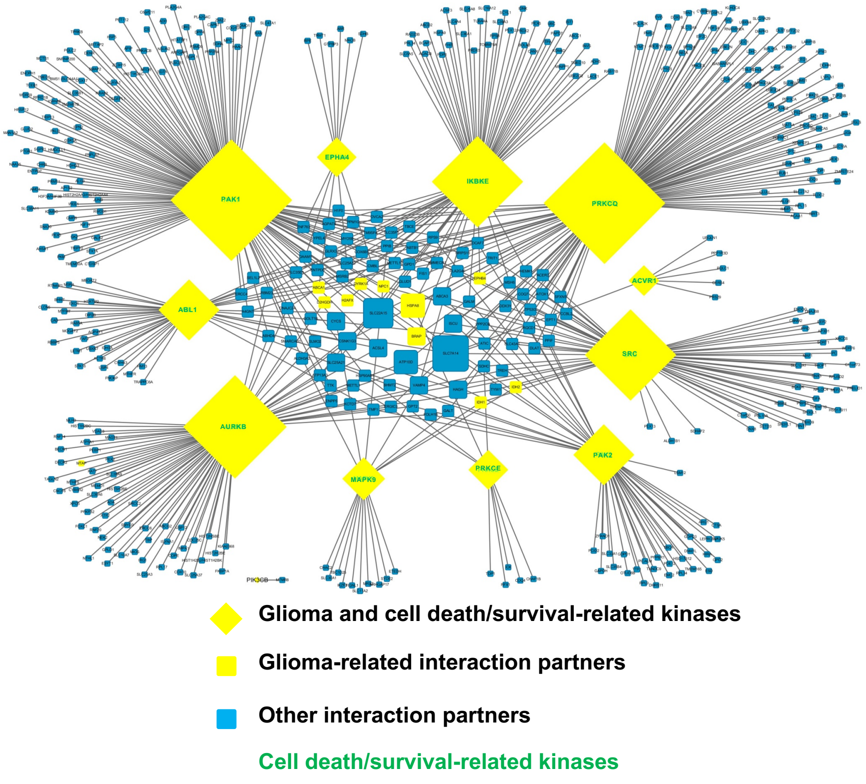

e Subnetwork of glioma, cell migration and cell death/survival-related kinases

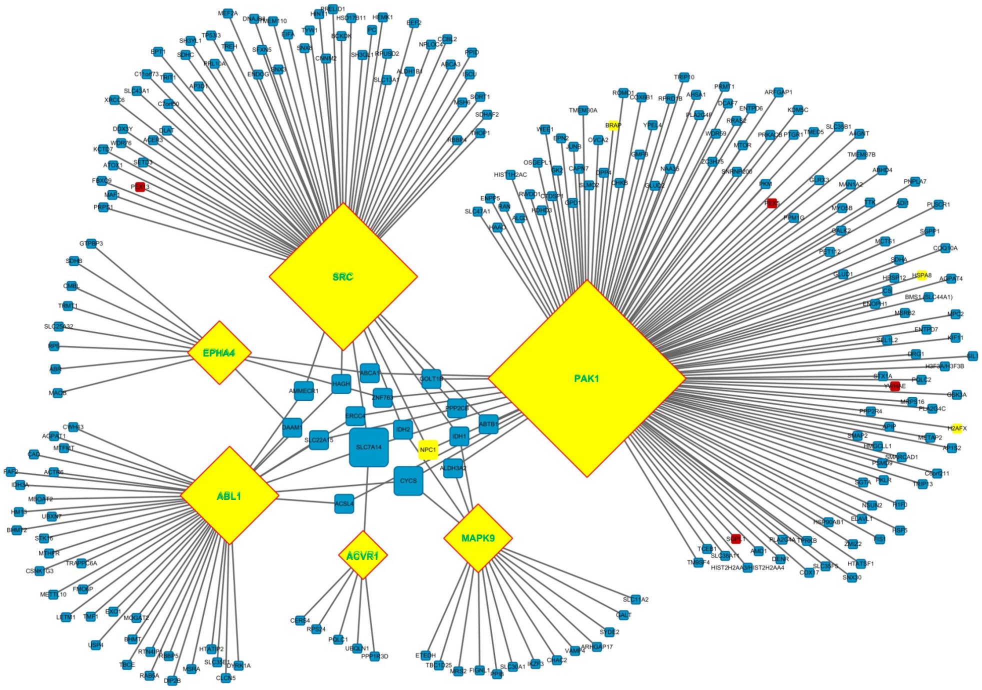

Glioma, cell migration and death/survival-related kinases

Cell migration-related interaction partners

Glioma-related interaction partners

Other interaction partners

Cell death/survival-related kinases

Figure S2. Subnetwork of human kinase-yeast genetic interactions. (a) Network of 28 kinases and their genetic interactions showing the relationships with cell migration and glioma. Subnetworks were also constructed with a focus on cell migration (b), glioma and cell migration (c), glioma and cell death/survival (d), and glioma, cell migration, and cell death/survival (e).
